# Types of Perception of Home Visiting Oral Health Care Services for Korean Older Persons: A Q Methodology Study

**DOI:** 10.3390/ijerph18010214

**Published:** 2020-12-30

**Authors:** Sue-Hyang Lee, Soo-Myoung Bae, Bo-Mi Shin, Sun-Jung Shin

**Affiliations:** 1 Department of Dental Hygiene, College of Dentistry, Gangneung-Wonju National University, Gangneung-si 25457, Korea; cactus92@naver.com (S.-H.L.); edelweiss@gwnu.ac.kr (S.-M.B.); purplebom@gwnu.ac.kr (B.-M.S.); 2 Research Institute of Oral Science, Gangneung-Wonju National University, Gangneung-si 25457, Korea

**Keywords:** dental hygienists, home care services, Korea, long-term care, older persons, oral health, Q-methodology

## Abstract

This study was conducted using the Q methodology to categorize Korean older persons’ subjective perceptions of home visiting oral health care services. Various opinions regarding home visiting oral health care services were collected based on related literature, and by conducting in-depth interviews with 12 people. Thirty-two statements were finally selected, and Q classification was applied. Based on data analysis with the PC-QUANL program, six factors (seven types) were derived, which accounted for 49.6% of the total variance. By comprehensive analysis of the types of subjective perceptions of home visiting oral health care services, the following two characteristics were identified. Korean older persons were expected to promote their own oral health activities, or improved access to expert health care services, through the home visiting oral health care services. Additionally, they had a need for social, economic, emotional, and informational support. Therefore, home visiting dental personnel should be able to provide customized visiting oral health care services based on evaluation of the need and type of perception of older persons. Thus, it is essential for visiting dental personnel to be trained in the knowledge of social welfare, and to develop diverse competencies.

## 1. Introduction

With an increase in the proportion of older persons in the population, a “healthy aging” policy is being emphasized to extend the healthy lifespan [1]. Good oral health of older persons is an essential requirement for ensuring “healthy aging” since it influences their ability to choose and digest food, thus, affecting their nutritional status and quality of life [2,3,4]. Moreover, oral infections and inflammatory reactions have been associated with non-infectious diseases [5,6,7]. In general, oral health problems appear cumulatively throughout life and tend to be amplified in old age [8], which may negatively affect both older persons and the national medical finances [9]. Thus, there is a need for a public health care approach in which local communities could manage the health of their own populations and proactively control medical expenses through early diagnosis via health check-ups [10].

In Korea, oral health care services are delivered to the vulnerable older population through the long-term care insurance program and home visiting health care programs. Via the long-term care insurance program, a caregiver can provide older persons with oral hygiene assistance. Alternatively, visiting nursing personnel, including dentists and dental hygienists (full-time personnel among doctors, oriental doctors, dentists belonging to long-term care institutions, nurses with more than 2 years of nursing experience, nursing assistants with more than 3 years of nursing assistant work experience, and dental hygienists) may visit the place where the older persons live, and provide professional oral health care and dental treatment that have been prescribed on the visiting nursing instructions (for dentists), and issued by the dentist, according to the visit cycle determined by the dentist. However, dental hygienist-delivered visiting oral health care services have been shown to be insufficient to date [11]. In the case of home visiting health care programs, a government-employed dental hygienist in charge of home visiting health care services may visit the homes of underprivileged families in the local community to provide oral health education (tooth brushing and denture management). However, due to lack of human resources, the delivery rate is decreasing [11,12]. To prepare for the upcoming super-aged society, the Korean government announced a community care policy in November 2018, which allows older persons to live healthier lives, in their older years, at their own homes. The visiting health care project is a key initiative in the community care policy and it is planned to expand the beneficiary households by 2025. It is a major project that the Korean Public Health Center has been conducting since the 1990s, and has been evaluated to have the effect of reducing medical expenses by improving the health care ability of older persons [13,14]. Therefore, it is necessary to initiate oral health care services that are not included in the public and health welfare policy for the older population [11].

According to an assessment of the effectiveness of visiting oral health care services for older persons, the tongue plaque, oral odor index, and oral bleeding were significantly reduced, and contributed to the improvement in oral hygiene management ability and quality of life [15,16]. In Australia, the involvement of experts who are aware of the importance of oral health and are able to conduct targeted and customized oral health care led to improvement in the oral health conditions of older persons and reduction in the requests for dental treatment, proving the effectiveness of dental experts’ intervention [17,18]. The need for visiting oral health care services in community care has been emphasized as a result of examining the oral health status and behavior of the targets of the older persons community care pilot project conducted in Cheonan in 2019. The number of existing natural teeth and the tooth surface bacterial membrane index were low, and the brushing practice rate and regular dental visit rates were found to be low [19].

In order to effectively operate a national policy or project, it was suggested that various perceptions of stakeholders be empirically reviewed [20] (pp. 52–87), and an ideal review method include both quantitative and qualitative information [21] (pp. 1–164). The reason for including qualitative information is that it can be used as basic data to identify the various experiences, attitudes, and thoughts of stakeholders, check their behavioral characteristics, and seek ways to develop policies and projects accordingly [22,23].

Q methodology is a complementary research methodology that can review both quantitative and qualitative information. The subjective perception of humans is objectified and measured scientifically, and it is possible to classify types and confirm the characteristics of types based on differences that appear differently between people [24] (pp. 78–191). In 2020, Australia will use the Q methodology to identify and compare priorities for the national older persons care, and stakeholders related to the national older persons care, namely, the older persons, dependent family members, and caregivers, to meet the expectations of older persons and their dependent family members, and to propose an approach and work behavior that can increase the work efficiency of caregivers [25,26].

Accordingly, in this study, we aimed to categorize the subjective perceptions of older persons regarding the visiting oral health care services for older persons and identify the characteristics of each type. The study results are intended to be used as basic data to determine ways to improve the home visiting oral health care services in South Korea.

## 2. Materials and Methods

**Figure 1:**
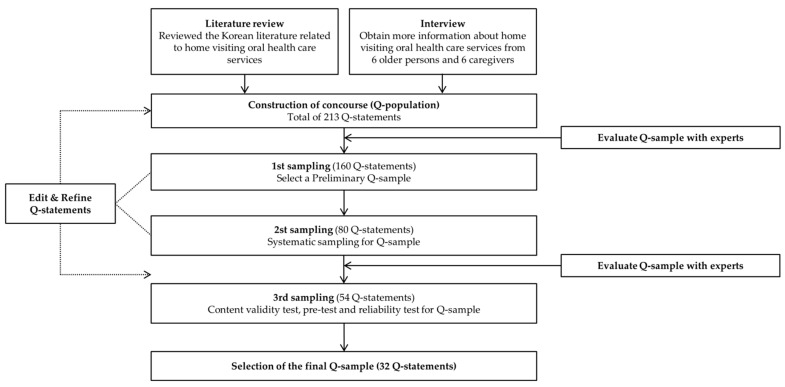
Flowchart of Q-sample construction.

The flowchart of the Q-sample construction and selection process is shown in Figure 1. This study was approved by the Bioethics Committee of Gangneung-Wonju National University (IRB no. GWNUIRB-2019-09).

### 2.1. Step 1: Collection of Opinion Statements and Selection of a Statement Set

The first step was establishing a Q-population. Q-statements collected through literature research and interviews that were written so as to inspire the subject’s awareness were included [27]. The literature search was conducted using four keywords: “older persons”, “oral health care”, “home visiting”, and “home care service” among Korean academic papers and dissertations published after 2010. From 32 previous studies, 148 statements including opinions on home visiting oral health care services were extracted. Furthermore, in-depth unstructured interviews were conducted with a total of 12 people: six caregivers and six older persons. The interviewers directly listened to and recorded the overall thoughts and requests of the interviewees, and extracted 65 additional statements [28,29].

Among the 213 statements, after removing duplicate statements, revising, and supplementing to prevent errors due to the subjectivity of the study participants, 160 statements that were believed to reflect the purpose of the study were selected. Subsequently, the 160 statements were categorized into economics, oral health, services, professionals, emotions, and others. In order to maintain the balance of the Q population, two researchers conducted systematic sampling in which statements were extracted proportionately from each category, and a total of 80 statements were selected by both researchers Among the 80 extracted statements, 54 Q-statements were selected after deleting or revising. The completed statements were reviewed and evaluated for grammar by a professional, with a Ph.D. degree in Korean language and literature; the statements with vague meaning were removed. Thereafter, an expert in Q methodology was consulted to evaluate any further problems in the methodology.

Finally, to select the Q-sample, a validity test, a pre-test, and a reliability test were conducted for the Q-statements. First, the content validity index was checked by seven experts to determine whether each Q-statement was representative of the Q-population (representativeness) and whether the content was appropriate (appropriateness) to confirm the participants’ subjective perception. For the 39 Q-statements with representativeness and appropriateness of 0.75 or higher, a pre-test was conducted on three people, which resulted in the selection of 32 Q-statements [30]. For these, a reliability test was conducted such that the Q-sort was performed among older persons and the general public twice with time differences. The average value of the correlation coefficient in this study was 0.775 [24], resulting in the confirmation of the 32 Q-statements as a Q-sample, as indicated in Table 1.

### 2.2. Step 2: Administration of the Q-Sort

To understand and identify the types of subjective viewpoints of older persons on home visiting oral health care services, older persons aged 65 years or more with different perspectives were selected as study subjects (P-sample). A total of 32 older persons living in Korea, who had varied characteristics in terms of sex, socioeconomic status, health status, and experience in using home visiting oral health care services, were randomly selected via a convenience sampling method [31]. However, older persons who had difficulty in classifying Q due to poor cognitive function and those who had been admitted to long-term care facilities for medical attention due to the spread of COVID-19 were excluded.

The Q-statements were allocated a number between 1 and 32 and printed on 148 × 105-mm cards in large letters for direct viewing and reading. Each participant read the 32 Q-sample cards and sorted them based on the level of agreement; though they agreed with were sorted on the right, those they disagreed with were sorted on the left, and neutral statements were sorted in the middle. Next, among the statements, they agreed with, the two cards they agreed with the most were placed at +4 and the rest were sorted toward the middle. Similarly, among the statements they disagreed with, the two cards they disagreed with the most were placed at −4 (Q-classification).

**Figure 2:**
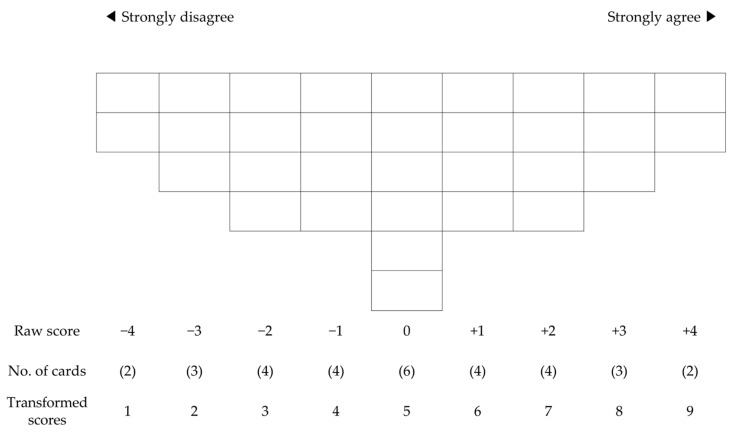
Score sheet used for ranking the statements.

The Q-response paper used in the study was configured to follow a curve of a normal distribution of −4 to +4 (Figure 2). For the four cards placed at both extremes (−4, +4), participants were interviewed about the reasons for such classification to facilitate subsequent analysis.

### 2.3. Step 3: Analysis and Interpretation of the Q-Sorts

The researcher collected data from June to July 2020.

The data organized in this way were subjected to principal components analysis using the PC-QUANL Program [32], and varimax rotation was used. Among the programs that analyze Q sort, the PC-QUANL Program extracts factors by maximizing the total variance, and shows differences between specific factors and other factors in addition to differences between factors; it is thus known as a program useful for distinguishing characteristics of factors that look similar [24,33]. The principal components analysis determines the number of factors based on the eigenvalue, and the minimum reference value is 1.0. Accordingly, to determine the older persons’ perception type of home visiting oral health care services, the number of factors was input from 3 to 6 based on an eigenvalue of 1.0 or higher. The number of factors was determined in consideration of the high total explanatory variation among the calculated results and the ease of interpreting the unique characteristics of each type.

Next, in order to interpret and name the characteristics of the recognition type, the following three were comprehensively reviewed and analyzed: (1) a statement with a standard score (Z-score) of ±1.00 or higher for the corresponding type; (2) a statement in which the difference between the standard score of the corresponding type (Z-score) and the average standard score (Z-score) of the other types was ±1.00 or more; and (3) demographic information and interview content of the participant with the highest factor weight in the corresponding type.

Finally, in order to minimize the researcher’s interpretation error on the characteristics of the perception type, a peer review method was conducted by three doctors with a high level of empirical and academic understanding of home visiting oral health care service to secure validity.

## 3. Results

The average time required for Q-classification and interviews was approximately 40 min. Among the 32 participants, there were 7 men and 25 women with a mean age of 79.9 (range 65–93) years. As shown in Table 2, seven Q-types of home visiting oral health care service perceptions were recognized among the participants. Following varimax rotation, six factors that explained 49.6% of the total variance were retained: Q-factor 1 (23.14%), Q-factor 2 (7.47%), Q-factor 3 (5.87%), Q-factor 4 (4.97%), Q-factor 5 (4.26%), and Q-factor 6 (3.84%). The eigenvalues were 7.4, 2.4, 1.9, 1.6, 1.4, and 1.2, respectively. Q-factor 2 formed Q-types II and Ⅶ. This is because types II and VII had a correlation coefficient of 0.029 between factors. In other words, since a correlation coefficient close to 0 indicates an independent relationship that is, items not related to each other, approximately 31% (4 out of 13) of the Q-sorts of older persons classified in Q-factor 2 were classified as type VII (Table 3).

### 3.1. Type I: Integrated Health Promotion Visiting Services Centered on Oral Health Education Type

Although the five type I older persons had low income and education levels in common, they would actively participate in community health care programs. As shown in Table 1, type I participants were found to agree the most with Q9 (”The reason that older persons neglected oral health management is that they did not receive proper oral health management education suitable for their oral health conditions”) and Q30 (“Dental personnel who provide home visiting oral health care services for the aged population must be able to intervene in the management of not only oral health but also systemic diseases”) and disagree the most with Q32 (“Older persons prefer a regular daily visit from dental personnel even if the visit time is short”). By comparing the average Z standard scores of different types, they agreed relatively more with Q28 (“Dental personnel visiting older persons’ homes should have enough conversation to be able to understand them”) and disagree with Q11 (“Older persons want to restore their confidence in their pronunciation and appearance through prosthetic treatments”) relatively more compared with other types. Therefore, they wished to receive a variety of health care services in one visit and preferred that visiting dental personnel have sufficient dialogue to understand them.

### 3.2. Type II: Independent Oral Health Management Practice Type

Type II consisted of nine older persons with a strong sense of independence. As shown in Table 1, they agreed the most with Q4 (“Oral health is very important in the life of older persons”) and Q7 (“It is important to timely prevent tooth or gum problems in older persons”) and disagreed the most with Q32 (“Older persons prefer a regular daily visit from dental personnel even if the visit time is short”). By comparing the average Z standard scores of different types, it was found that they agreed with Q26 (“It is very important for older persons to establish a trustworthy relationship with the dental personnel visiting their home”) and disagreed with Q30 (“Dental personnel who provide home visiting oral health care services for the aged population must be able to intervene in the management of not only oral health but also systemic diseases”) and Q8 (“The state should supply oral health care products free of charge so that older persons can manage oral hygiene well”) relatively more compared with the other types. In other words, they desired preventive oral health care services under an intensive, systematic, and reliable system for the older persons without burdening the state and dental personnel. Moreover, instead of being provided oral health care products or services for free, they thought it was desirable that older persons pay a minimum cost so that they can take responsibility for their own dental health.

### 3.3. Type III: Free Home Visiting Oral Health Care Service Proponent Type

The three older persons who fell under type III were indifferent to oral health prevention due to economic difficulties, accumulated household medical expenses, and lacked oral health awareness. As shown in Table 1, type III participants agreed the most with Q3 (“The cost of home visiting oral health care services for the aged population must be paid by the state”) and Q8 (“The state should supply oral health care products free of charge so that older persons can manage oral hygiene well”) and disagreed the most with Q32 (“Older persons prefer a regular daily visit from dental personnel even if the visit time is short”). In the comparison of the average Z standard scores of different types, they agreed with Q3 and Q8, which was the most agreeable item, mentioned earlier, much more than did other types. On the other hand, they disagreed with Q7 (“It is important to timely prevent tooth or gum problems in older persons”). Given the items of agreement and disagreement in the Q-survey, they hoped for unconditional support of the state for home visiting oral health care services.

### 3.4. Type IV: Complete Oral Health Value Pursuit Type

One older person corresponding to type IV agreed the most with Q21 (“Older persons want to receive oral hygiene management from a professional dental hygienist rather than their family members or caregivers”) and Q29 (“Dental personnel visiting older persons’ homes should help relieve oral pain and discomfort from chewing so that older persons can consume foods (nutrients) of various textures”) and disagreed the most with Q5 (“Despite the sore teeth and discomfort on chewing, older persons believe it does not interfere with their lives”) and Q32 (“Older persons prefer a regular daily visit from dental personnel even if the visit time is short”). The comparison of the average Z standard scores of different types showed that the type IV participant agreed with Q11 (“Older persons want to restore their confidence in their pronunciation and appearance through prosthetic treatments”). Moreover, they disagreed with Q26 (“It is very important for older persons to establish a trustworthy relationship with the dental personnel visiting their home”) relatively more compared with the other types. In other words, this older persons wanted to improve oral function and restore pronunciation and appearance through oral health management services of dental personnel.

### 3.5. Type V: Dental Hygienist-Led Diversified Oral Health Care Service Demand Type

As seen in Table 1, type V agreed the most with Q24 (“It is necessary that dental hygienists regularly visit older persons for the prevention and management of oral diseases while providing them with appropriate oral health care services”) and Q25 (“Dental hygienists must visit older persons’ homes and deliver oral health care services, and if dental treatment is needed, they must also provide a transfer service to a dental clinic and accompany them”) and disagreed the most with Q2 (“The cost of home visiting oral health care services should be determined by reflecting the level of expertise and work intensity of visiting dental personnel rather than the economic level of the older population”) and Q3 (“The cost of home visiting oral health care services for the older population must be paid by the state”). In the comparison of the average Z standard scores of different types, they found Q5 (“Despite the sore teeth and discomfort on chewing, older persons believe it does not interfere with their lives”), Q24 (“It is necessary that dental hygienists regularly visit older persons for the prevention and management of oral diseases while providing them with appropriate oral health care services”), and Q25 (“Dental hygienists must visit older persons’ homes and deliver oral health care services, and if dental treatment is needed, they must also provide a transfer service to a dental clinic and accompany them”) much more agreeable and they disagreed with Q19 (“Home visiting oral health care services should ensure adequate and safe oral health care for the older persons with systemic diseases or disabilities”) relatively more compared with the other types. In other words, they hoped that dental hygienist would visit them regularly to provide social support, in addition to oral health care services, because they felt that older persons in general are insensitive to their oral health status. Moreover, they believed that the related services should be charged a realistic fee, which would take into account the national finances and the expertise and workload of the professionals rather than the unconditional support of the state.

### 3.6. Type VI: Comprehensive Health and Oral Health Care Service Demand Type

As shown in Table 1, type VI participants agreed the most with Q30 (“Dental personnel who provide home visiting oral health care services for the aged population must be able to intervene in the management of not only oral health but also systemic diseases”) and disagreed the most with Q2 (“The cost of home visiting oral health care services should be determined by reflecting the level of expertise and work intensity of visiting dental personnel rather than the economic level of the older population”). By comparing the average Z standard scores of different types, it was found that they agreed with Q30, which was the most agreeable item, and disagreed with Q3 (“The cost of home visiting oral health care services for the aged population must be paid by the state”) much more than did the other types. In other words, they were willing to use the home visiting services at a minimum cost if the dental personnel could provide comprehensive intervention for systemic diseases along with oral health care.

### 3.7. Type VII: Home Visiting Oral Health Care Service through Emotional Connection Type

Type VII older persons were recipients of long-term care benefits or participants in community health promotion programs. They had a high degree of trust and adherence to professional personnel and tended to be extroverts who enjoyed being visited in their homes. As shown in Table 1, type VII participants strongly agreed with Q29 (“Dental personnel visiting older persons’ homes should help relieve oral pain and discomfort from chewing so that older persons can consume foods (nutrients) of various textures”) and Q30 (“Dental personnel who provide home visiting oral health care services for the aged population must be able to intervene in the management of not only oral health but also systemic diseases”) and disagreed the most with Q20 (“Home visiting oral health care services should deliver the services older persons want rather than those determined to be necessary by dental personnel”) and Q31 (“Older persons feel uncomfortable with oral hygiene by opening their mouths to non-family members”). The comparison with the average Z standard scores of other types show that they thought positively of Q32 (“Older persons prefer a regular daily visit from dental personnel even if the visit time is short”), the item that was relatively disagreeable by most types and found Q20 very disagreeable, which was similar to the aforementioned findings. In other words, they desired to be able to eat a variety of foods and improve their overall health with the help of the visiting dental personnel. Furthermore, they highly trusted the services provided by the experts and very much preferred daily visits, even for a short time.

### 3.8. Consensus Items and Average Z-Score

All seven different types derived from this study agreed with Q13 (“Older persons do not know the procedure for using home visiting oral health care services”) (Z = 0.66). In other words, most Korean older persons do not know how to use home visiting oral health care services.

## 4. Discussion

In this study, the Q methodology was used to identify the types and characteristics of older persons’ perception of home visiting oral health care services in order to propose practical solutions considering older persons-centered visiting oral health care service items and the role of providers. Depending on their health locus of control, Korean older persons were expected to promote their own oral health activities, or improved access to expert health care services, through the home visiting oral health care services. Additionally, they had a need for social, economic, emotional, and informational support.

Q methodology is a hypothetical abductive methodology that gradually uncovers hypotheses [34]. It is a research methodology that starts from the participant’s point of view, and not from the researcher’s assumptions, to “create hypotheses” and “discover hypotheses” about the research topic. Accordingly, the following four behavioral characteristics were found as a result of comprehensively interpreting the types and characteristics of each type of perception of Korean older persons for home visiting oral health care services.

First, older persons want to practice self-directed oral health promotion through home visiting oral health care services provided during regular visits of dental personnel. This can be explained through types I, II, and IV. Among the three types, the five type I older persons with the highest explanatory power were all participants in community health care programs. It has been reported that those who had prevention and management experiences through community health care programs or health check-ups had a higher rate of health-promoting behaviors since they believed they could complete the practices successfully by receiving periodic encouragement, support, and information tailored to their lifestyles [35,36]. These people tend to have higher self-efficacy than those who do not have such a support system. In general, health-promoting behavior is affected by the social support network [37] and health locus of control [38] in addition to self-efficacy [39]. Among them, the internal health locus of control, which refers to individual expectations and beliefs about what their health depends on, is known as the major factor in predicting health-promoting behavior [40,41,42]. Hence, it is necessary to continuously expand the areas where older persons can feel a sense of accomplishment by slowly starting with simple actions, such as oral cleaning, in order to enhance their self-efficacy and their internal health locus of control. In addition, in the process of delivering customized home visiting oral health care services to the older population, dental professionals should provide older persons with training to strengthen their competence so that they can improve self-efficacy through continuous encouragement and social support [43].

Second, older persons who are financially underprivileged show a passive tendency toward all oral health care services that incur costs. This is explained through type III. In addition, less awareness of the importance of oral health can lead to deepened oral health inequality. Therefore, oral health management education must be given a priority so that older persons understand the correlation between oral and systemic diseases and recognize the importance of preventive care. Thereafter, oral health care products suitable for older persons should be delivered free of charge to encourage habituation of dental care, thereby, establishing trust and relationship with the target subjects.

Third, older persons with weak social support networks have high demands for comprehensive and multifaceted health care services from experts at a minimal cost (types V and VI), and those who are highly social, want to receive comprehensive oral health care services through emotional connections based on regular daily visits by dental personnel (type VII). In general, older persons who live alone have a weak social support network, complex physical, economic, psychological, and social problems, and more difficulties in maintaining their daily life independently and living safely than the general older population [44,45]. Therefore, a welfare plan for resolving oral health inequality base on the income level and type of residence must be considered [46,47]. Moreover, the sense of belonging and self-integration in old age—that is, social support, is a very important factor in increasing the sense of happiness of older persons and a major determinant of health behavior [48,49]. In Spain, home care services are not only limited to visiting services but also include welfare services, such as a phone service for older persons who may be feeling lonely [50]. Hence, dental personnel who visit older persons’ homes should evaluate their social and emotional support needs and determine the frequency of visits accordingly.

Fourth, it was found that most Korean older persons perceived that older adults do not know how to use home visiting oral health care services. Thus, it is essential to market the program through mass media that are easily accessible to the older population.

This study has a few limitations. We failed to include the perceptions of caregivers and dental personnel that could affect the use of home visiting oral health care. However, it is significant as the first study that applied the Q methodology to categorize the subjective perception of beneficiaries (older persons) about home visiting oral health care services and confirm the characteristics of each type. In particular, it was confirmed that the older persons in South Korea were positively aware of the operation and activation of visiting oral health care services. Therefore, practical discussions should be made for the establishment of an advanced home visiting oral health care service that reflects the characteristics of various types of perceptions of older persons. For example, the development of multifaceted service items should be discussed so that the various needs of older persons identified in the research results can be satisfied. In addition, a systematic assessment tool needs to be developed to accurately identify the needs of older persons and deliver appropriate services. Based on this, in the long term, it is necessary for the home visiting oral health care service to provide psychological and social care for older persons in addition to dental medical intervention, thereby establishing the value and meaning for older persons to practice oral health behavior on their own.

## 5. Conclusions

From the examined hypotheses, it can be concluded that it is necessary to establish a more comprehensive role for dental personnel in order to provide customized home visiting oral health care services for older persons in preparation for a super-aged society. In other words, in addition to the professional oral health management they are performing, dental personnel should be provided professional competency development and continuous training to strengthen the internal control and self-efficacy of older persons who can continue their oral health-promoting activities. Moreover, a comprehensive and integrated approach is warranted that can support timely dental treatment and regular prevention by helping older persons recognize the association between oral diseases and underlying health conditions and provide dental personnel with training in social and welfare knowledge.

## Figures and Tables

**Table 1:** Statement rank scores.

Statement		Z-Score ^1^						
		Ⅰ	Ⅱ	Ⅲ	Ⅳ	Ⅴ	Ⅵ	Ⅶ
1	Older persons give up dental treatment even if they need it due to financial difficulties.	−0.6	−0.6	1.0	0.0	1.0	0.4	1.5
2	The cost of home visiting oral health care services should be determined by reflecting the level of expertise and work intensity of visiting dental personnel rather than the economic level of the older population.	−0.8	−0.6	0.3	0.0	−1.6	−1.7	−1.0
3	The cost of home visiting oral health care services for older persons must be paid by the state.	−0.4	−1.3	1.9	−0.9	−1.5	−1.6	1.1
4	Oral health is very important in the life of older persons.	1.4	2.4	0.7	0.9	0.8	1.0	0.8
5	Despite the sore teeth and discomfort on chewing, older persons believe it does not interfere with their lives.	−1.4	−1.1	−0.9	−1.8	0.8	−1.7	−0.9
6	Older persons prefer on-site dental treatment via home visiting oral health care services.	−1.8	0.1	−1.7	−0.9	−0.8	0.0	−0.8
7	It is important to timely prevent tooth or gum problems in older persons.	1.4	2.2	−0.7	1.4	1.5	1.6	1.7
8	The state should supply oral health care products (toothbrush, interdental toothbrush, denture cleaner, etc.) free of charge, so that older persons can manage oral hygiene well.	1.2	−1.5	1.9	0.9	−1.4	0.1	−0.1
9	The reason that older persons neglected oral health management is that they did not receive proper oral health management education suitable for their oral health conditions.	1.5	0.7	−0.4	1.4	0.9	1.3	−0.9
10	An oral examination system should be established in which dental personnel would regularly visit older persons in their homes to check their oral health.	0.0	−1.1	0.3	−0.5	1.2	0.4	0.7
11	Older persons want to restore their confidence in their pronunciation and appearance through prosthetic treatments (dentures, implants, etc.).	−1.6	−0.7	−0.3	1.4	0.1	−0.9	−1.2
12	Dental personnel should be in charge of dental health care for older persons with dementia.	−1.2	−0.8	−0.8	0.5	−0.8	−0.0	−0.8
13	Older persons do not know the procedure for using home visiting oral health care services.	0.8	0.8	0.5	0.5	0.9	0.6	0.6
14	Unless continuous quality management of home visiting oral health care services for older persons is assured, it is difficult to stimulate these services.	−0.9	0.3	−0.3	0.0	−1.1	−1.2	−0.8
15	During the home visiting oral health care service, meals appropriate to the older persons’ oral conditions, or nutritional guidance should be provided.	0.4	−0.2	−0.8	−0.5	−0.7	−0.3	−0.8
16	In order to improve the oral health of older persons, the number of dental personnel visiting their homes and providing oral health care services should increase in the future.	0.2	−0.1	1.9	0.0	0.8	−0.8	−0.1
17	Dental hygienists must be able to accurately identify the oral health care needs and provide the necessary oral health care services to older persons.	0.3	0.3	−0.0	−1.4	0.8	−0.3	−0.1
18	South Korea lacks high-quality oral health care services that can meet the expectations of older persons.	−0.4	−0.1	0.6	−1.4	0.1	0.3	−0.6
19	Home visiting oral health care services should ensure adequate and safe oral health care for older persons with systemic diseases or disabilities.	0.6	0.6	1.2	0.5	−0.7	−0.6	0.8
20	Home visiting oral health care services should deliver the services older persons want rather than those determined to be necessary by dental personnel.	−0.7	0.5	−0.0	0.9	−1.3	0.9	−1.9
21	Older persons want to receive oral hygiene management from a professional dental hygienist rather than their family members or caregivers.	0.8	1.0	0.6	1.8	1.1	0.6	0.7
22	To improve the oral health of older persons, dentists must visit their homes to conduct oral examinations, and dental hygienists must visit them regularly and provide oral health care services according to the results of the examinations.	−0.7	−0.6	−0.7	0.0	−1.0	0.4	0.8
23	Dental hygienists can play an important role in ensuring that older persons have correct oral health care behaviors.	0.4	0.4	−0.7	0.5	−0.9	−1.3	−0.8
24	It is necessary that dental hygienists regularly visit older persons for the prevention and management of oral diseases while providing them with appropriate oral health care services.	−0.6	−0.6	0.6	−0.5	1.7	0.8	0.5
25	Dental hygienists must visit older persons’ homes and deliver oral health care services, and if dental treatment is needed, they must also provide a transfer service to a dental clinic and accompany them.	−0.8	−0.3	−1.3	0.9	1.6	1.6	0.3
26	It is very important for older persons to establish a trustworthy relationship with the dental personnel visiting their home.	0.2	1.4	0.4	−1.4	0.5	−0.4	−0.6
27	Dental personnel visiting older persons’ homes should have no difficulty communicating with them.	0.6	0.2	−1.0	−0.9	0.2	0.0	0.9
28	Dental personnel visiting older persons’ homes should have sufficient conversation to be able to understand them.	1.0	0.9	−0.9	−0.9	−0.9	0.2	0.0
29	Dental personnel visiting older persons’ homes should help relieve oral pain and discomfort from chewing so that older persons can consume foods (nutrients) of various textures.	1.1	0.1	0.8	1.8	0.8	0.7	1.7
30	Dental personnel who provide home visiting oral health care services for older persons must be able to intervene in the management of not only oral health but also systemic diseases (diabetes, hypertension, etc.).	1.5	−1.5	0.4	−0.5	−0.6	2.1	1.7
31	Older persons feel uncomfortable with oral hygiene by opening their mouths to non-family members.	0.4	0.7	0.0	0.0	−0.9	−0.7	−1.8
32	Older persons prefer a regular daily visit from dental personnel even if the visit time is short.	−2.0	−1.9	−2.4	−1.8	−0.9	−1.6	−0.5

^1^ Z-score: value rounded to decimal places.

**Table 2:** General characteristics and factor weights of P-samples by step.

Q Type	ID (Factor Weight)	Demographic Variable								
		Age	Sex	Edu ^1^	Income	Living Alone	Comorbidity	Dental Prosthesis	Invalidity	Classification
Ⅰ	16 (1.1223)	83	F	Un	Low	Yes	Yes	Full dentures	Yes	CHCP ^2^
	17 (0.1257)	80	F	Un	Low	Yes	Yes	Full dentures	Yes	CHCP ^2^
	18 (0.2339)	86	M	Un	Low	No	No	Full dentures	No	CHCP ^2^
	22 (0.4492)	79	F	Un	Low	No	No	None	No	CHCP ^2^
	27 (0.5525)	82	F	Un	Low	No	No	Full dentures	Yes	CHCP ^2^
Ⅱ	1 (0.5529)	84	F	E	Low	Yes	No	None	No	NBLS ^3^
	3 (0.8268)	74	F	H	Low	No	No	One implant	No	None
	9 (0.5375)	93	F	Un	Low	Yes	No	Full dentures	No	NBLS ^3^
	12 (0.5992)	69	F	U	Low– middle	No	No	None	No	CHCP ^2^
	23 (0.2380)	73	M	H	Middle –high	Yes	No	Full dentures	No	CHCP ^2^ PV ^4^
	24 (0.9733)	80	F	M	Low– middle	No	No	Full dentures	No	CHCP ^2^
	28 (0.2094)	88	M	Un	Low	No	No	Full dentures	Yes	CHCP ^2^
	29 (0.2162)	81	M	Un	Low	No	No	None	No	CHCP ^2^
	31 (0.4031)	84	F	M	Low	No	Yes	Full dentures	Yes	LHC ^5^
Ⅲ	6 (0.6711)	91	F	Un	Low	Yes	No	Full dentures	No	None
	19 (0.9348)	65	F	E	Low	No	No	None	No	CHCP ^2^
	30 (0.6178)	75	F	Un	Low	No	Yes	Partial denture	No	CHCP ^2^
Ⅳ	2 (0.9991)	84	F	Un	Low– middle	Yes	No	None	No	None
Ⅴ	5 (0.4575)	87	F	Un	Low	Yes	No	Full dentures	Yes	NBLS ^3^
	14 (0.3249)	79	F	Un	Low	Yes	No	None	No	CHCP ^2^
	15 (0.4428)	73	M	H	Low	Yes	No	Full dentures	No	CHCP ^2^
	25 (0.8335)	72	F	M	Low	Yes	No	Two implants	No	CHCP ^2^
	26 (0.4269)	71	M	M	Low– middle	No	No	Seven implants	No	CHCP ^2^
Ⅵ	7 (0.3552)	83	F	Un	Low	Yes	Yes	Full dentures	No	NBLS ^3^
	10 (0.4728)	82	F	Un	Low	Yes	No	Partial denture	No	None
	11 (0.2298)	79	F	Un	Low	Yes	No	Partial denture	No	NBLS ^3^
	20 (0.5959)	73	F	M	Middle –high	Yes	No	Maxillary denture	No	CHCP ^2^
	21 (0.3468)	81	F	Un	Low	Yes	No	Full dentures	No	CHCP ^2^
Ⅶ	4 (0.1948)	88	F	Un	Low	Yes	Yes	Full dentures	Yes	NBLS ^3^ LHC ^5^
	8 (0.4485)	85	F	Un	Low	No	No	Full dentures	Yes	LHC ^5^
	13 (0.0779)	76	M	U	Low– middle	No	No	Seven implants	No	CHCP ^2^
	32 (0.3326)	76	F	Un	Low	Yes	No	Maxillary denture	Yes	NBLS ^3^ LHC ^5^

^1^ Educational level: Un (uneducated persons), E (elementary school), M (middle school), H (high school), U (university) graduation ^2^ CHCP: Participants in Community Health Care Programs managed by Gangneung-si or Pyeongchang Public Health Center. They receive exercise, nutrition, and chronic disease management through community health care programs. ^3^ NBLS: National Basic Livelihood Security recipients-persons whose income recognized amount is 30–50% or less of the median income, which is less than the minimum cost of living. They receive support from the state for living expenses divided into livelihood, medical, housing, and education. ^4^ PV: Patriots and Veterans-persons who have contributed or sacrificed for the state. The state pays them veterans’ benefits and provides them with reductions and exemptions in education, employment, and medical care. ^5^ LHC: Long-term Home Care recipients-older persons who use home-based care services through regular visits by caregivers under the Korean Long-Term Care Insurance System.

**Table 3:** Correlations between types.

Type	Type Ⅰ	Type Ⅱ	Type Ⅲ	Type Ⅳ	Type Ⅴ	Type Ⅵ	Type Ⅶ
**Type Ⅰ**	1.000	0.510	0.412	0.379	0.225	0.505	0.445
**Type Ⅱ**	0.510	1.000	0.009	0.339	0.315	0.307	0.029
**Type Ⅲ**	0.412	0.009	1.000	0.243	0.057	0.030	0.313
**Type Ⅳ**	0.379	0.339	0.243	1.000	0.203	0.439	0.162
**Type Ⅴ**	0.225	0.315	0.057	0.203	1.000	0.492	0.407
**Type Ⅵ**	0.505	0.307	0.030	0.439	0.492	1.000	0.466
**Type Ⅶ**	0.445	0.029	0.313	0.162	0.407	0.466	1.000

## Data Availability

The data presented in this study are available on request from the corresponding author. The data are not publicly available because it reflects the subject’s personal experience and subjective thoughts.

## References

[1] Swedish National Institute of Public Health Healthy Ageing: A Challenge for Europe. http://www.healthyageing.eu/?q=resources&page=10.

[2] Glick M., Williams D.M., Kleinman D.V., Vujicic M., Watt R.G., Weyant R.J. (2016). A new definition for oral health developed by the FDI World Dental Federation opens the door to a universal definition of oral health. Br. Dent. J..

[3] Rouxel P., Tsakos G., Chandola T., Watt R.G. (2016). Oral Health-a neglected aspect of subjective wellbeing in later life. J. Gerontol. B Psychol. Sci. Soc. Sci..

[4] Walls A.W., Steele J.G., Sheiham A., Marcenes W., Moynihan P.J. (2000). Oral health and nutrition in older people. J. Public Health Dent..

[5] Lam O.L., Zhang W., Samaranayake L.P., Li L.S., McGrath C. (2011). A systematic review of the effectiveness of oral health promotion activities among patients with cardiovascular disease. Int. J. Cardiol..

[6] Locker D. (1988). Measuring oral health: A conceptual framework. Commun. Dent. Health.

[7] Murray Thomson W. (2014). Epidemiology of oral health conditions in older people. Gerodontology.

[8] Millar W.J., Locker D. (1999). Dental insurance and use of dental services. Health Rep..

[9] Christian B., Chattopadhyay A. (2014). Determinants and trends in dental expenditures in the adult US population: Medical Expenditure Panel survey 1996–2006. Commun. Dent. Health.

[10] Kim J.K. (2014). A study on senior human rights in an aging society. J. Soc. Welf. Manag..

[11] Lee S.H., Bae S.M., Shin B.M., Shin S.J. (2020). Current status and future tasks of visiting oral health care services for elders. J. Korean Soc. Dent. Hyg..

[12] Lee G., Yang S.J., Woo E. (2018). Past, present, and future of home visiting healthcare services based on public health centers in Korea. J. Korean Pubilc Health Nurs..

[13] Kim J.H., Lee T.J., Lee J.H., Shin S.J., Lee E.H. (2010). A cost benefit analysis of individual home visiting health care. J. Korean Acad. Commun. Health Nurs..

[14] Ko Y., Lee I.S. (2011). Cost–benefit analysis of home visiting care for vulnerable populations with hypertension. J. Korean Acad. Commun. Health Nurs..

[15] Bok H.J. (2019). Evaluation on Home Visiting Oral Health Program for the Elderly in Rural Community. Ph.D. Thesis.

[16] Roh J.Y. (2014). The Evaluation on the Home Visiting Oral Health Program for 4 Years at Chil-Gok County. Master’s Thesis.

[17] Low L.F., Yap M., Brodaty H. (2011). A systemic review of different models of home and community care services for older persons. BMC Health Serv. Res..

[18] Wright F.C., Law G., Chu S.K.Y., Cullen J.S., Le Couteur D.G. (2017). Residential age care and domiciliary oral health services: Reach-OHT-The development of a metropolitan oral health programme in Sydney, Australia. Gerodontology.

[19] Jang J.H., Cho J.W., Kim Y.J., Ki J.Y., Jo K.S., Kim J.R., Park J.E., Kim D.H. (2020). Preliminary study for the development of a visiting oral health care intervention program for the elderly based on community healthcare. J. Korean Acad. Oral. Health.

[20] Goggin M.L. (1987). Policy Design and the Politics of Implementation: The Case of Child Health Care in the American States.

[21] Posavac E.J. (2015). Program. Evaluation: Methods and Case Studies.

[22] Fortune A.E., Reid W.J., Miller R.L. (2013). Qualitative Research in Social Work.

[23] Kweon Y.R., Kim H.S., Yoo B.N., Kim Y.S., Lee M.J. (2018). Qualitative analysis of ICT based health care, management for chronic disease patients. J. Korean Pubilc Health Nurs..

[24] Kim H.K. (2008). Q Methodology: Philosophy, Theories. Analysis and Application.

[25] Ludlow K., Churruca K., Mumford V., Ellis L.A., Braithwaite J. (2020). Staff members’ prioritisation of care in residential aged care facilities: A Q methodology study. BMC Health Serv. Res..

[26] Ludlow K., Churruca K., Ellis L.A., Mumford V., Braithwaite J. (2020). Family members’ prioritisation of care in residential aged care facilities: A case for individualised care. J. Clin. Nurs..

[27] Hughes M.J. (2012). Researching Behaviour: A Q Methodological Exploration of the Position of the Young Person as Researcher. Ph.D. Thesis.

[28] Garrido Urrutia C., Romo Ormazábal F., Espinoza Santander I., Medics Salvo D. (2012). Oral health practices and beliefs among caregivers of the dependent elderly. Gerodontology.

[29] Oh J., Yi M. (2012). Factors that affect health-related quality of life in community-dwelling older adults. Perspect. Nurs. Sci..

[30] Kim H., Kim J.W., Heo J.H., Kim D.Y., Sung S.J. (2008). Content validity of aphasia screening test protocol. Commun. Sci. Disord..

[31] Stephenson W. (1952). Q-methodology and the projective techniques. J. Clin. Psychol..

[32] Akhtar-Danesh N., Baumann A., Cordingley L. (2008). Q-methodology in nursing research: A promising method for the study of subjectivity. West. J. Nurs. Res..

[33] Kim S.E. (2016). Q Methodology and Social Sciences.

[34] Stephenson W. (1982). Q-methodology, interbehavioral psychology, and quantum theory. Psychol. Rec..

[35] Park S.Y. (2018). Factors affecting the rate of oral examination in the elderly in local communities. J. Korean Soc. Dent. Hyg..

[36] Seo H.M., Hah Y.S. (2004). A study of factors influencing on health promoting lifestyle in the elderly—Application of Pender’s health promotion model. J. Korean Acad. Nurs..

[37] Staniute M., Brozaitiene J., Bunevicius R. (2013). Effects of social support and stressful life events on health-related quality of life in coronary artery disease patients. J. Cardiovasc. Nurs..

[38] Bergvik S., Sørlie T., Wynn R. (2012). Coronary patients who returned to work had stronger internal locus of control beliefs than those who did not return to work. Br. J. Br. J. Health Psychol..

[39] Kang Y.H. (2013). Health promoting lifestyle, self-efficacy, and life satisfaction of middle-aged women. J. Korean Data Anal. Soc..

[40] Grotz M., Hapke U., Lampert T., Baumeister H. (2011). Health locus of control and health behaviour: Results from a nationally representative survey. Psychol. Health Med..

[41] Waller K.V., Bates R.C. (1992). Health locus of control and self-efficacy beliefs in a healthy elderly sample. Am. J. Health Promot..

[42] Wallston K.A., Wallston B.S., DeVellis R. (1978). Development of the multidimensional health locus of control (MHLC) scales. Health Educ. Monogr..

[43] Nied R.J., Franklin B. (2002). Promoting and prescribing exercise for the elderly. Am. Fam. Physician.

[44] Choi Y. (2008). Economic and health status, social support and depression of the elderly living alone. Soc. Sci. Res. Rev..

[45] Jennifer Yeh S.C., Lo S.K. (2004). Living alone, social support, and feeling lonely among the elderly. Soc. Behav. Pers. Int. J..

[46] Kim S.M., Shin H. (2015). The effect of economic factors on private health insurance enrollment and dental care utilization. J. Korean Acad. Oral Health.

[47] Lee S.J., Shim M.S. (2020). Effects of health literacy and unmet health care needs on health promotion behavior among elderly in the community. J. Korean Pubilc Health Nurs..

[48] Orkibi H., Ronen T., Assoulin N. (2014). The subjective well-being of Israeli adolescents attending specialized school classes. J. Educ. Psychol..

[49] Wang W., Lau Y., Chow A., Thompson D.R., He H.G. (2014). Health-related quality of life and social support among Chinese patients with coronary heart disease in mainland China. Eur. J. Cardiovasc. Nurs..

[50] Aceros J.C., Cavalcante M.T.L., Domènech M. (2016). Aging at home with telecare in Spain. A dicourse analysis. Ciência Saúde Coletiva.

